# Epidemiology of functional gastrointestinal disorders using ROME III adult questionnaire, a population based cross sectional study in Karachi—Pakistan

**DOI:** 10.1371/journal.pone.0268403

**Published:** 2022-06-13

**Authors:** Shahab Abid, Hareem Rehman, Safia Awan, Azmina Artani, Imran Siddiqui

**Affiliations:** 1 Department of Medicine, Section of Gastroenterology, Aga Khan University, Karachi, Pakistan; 2 Department of Pathology and Lab Medicine, Aga Khan University, Karachi, Pakistan; Chiba Daigaku, JAPAN

## Abstract

**Objective:**

Functional Gastrointestinal Disorder (FGIDs) are a heterogenous group of disorders, with Irritable Bowel Syndrome (IBS) and Functional Dyspepsia (FD) being the most common disorders worldwide. The purpose of this study was to identify the spectra of FGIDs classified according to the ROME III criteria amongst an adult Pakistani population. It also aimed to correlate the psychosocial alarm symptoms with the prevalence of FGIDs and report the overlap of all FGID.

**Design:**

This was a community based cross-sectional study. Multi-stage cluster sampling technique was applied, and 1062 households were initially randomly chosen using systematic sampling technique. Only one person from each household was enrolled in the study. After eligibility screening, 860 participating individuals were requested to fill out a structured ROME III interview questionnaire, administered to them by a trained interviewer.

**Results:**

FGIDs were diagnosed in 468 individuals (54.4%), out of 860 participants. FD was found to be the most prevalent (70.2%), followed by Functional Heartburn (58.9%) and Functional bloating (56.6%). Amongst a total of 468 participants diagnosed with FGIDs, 347 (74.1%) had overlapping disorders. There was also a higher incidence of psychosocial alarm symptoms including higher pain severity (62.6% vs 46.4%) and being victimized at some point in their lives (26.1% vs 6.6%) amongst FGID patients.

**Conclusion:**

There is a high disease burden of FGIDs in this study population, with approximately half of the population suffering from at least one type of FGID. Overlapping disorders are also common in this part of the world.

## Introduction

Functional GI disorders (FGIDs) are a heterogenous group of disorders which include the Irritable bowel syndrome (IBS), functional abdominal bloating, functional constipation (FC), functional diarrhea (FD) and unspecified functional bowel disorder. Patients with FGIDs have chronic and recurrent symptoms, with a significant overlap amongst different disorders [1]. Despite popular belief that FGIDS are not attributable to any biochemical or structural etiology [2]. Recent researches have proven that certain biological mechanisms such as gut-brain dysfunction, chronic infections, genetic and environmental factors including diet, gut microbiota and use of antibiotics influence the development of these disorders [3–5]. The Rome criteria, established in 1992, became the primary diagnostic tool for FGIDs, used by physicians across the globe. Since there was an absence of chemical, physiological or radiological abnormalities, this criterion classified the disorders based on the patient’s symptoms. The disorders are broadly classified into six domains; Esophageal, Gastroduodenal, Bowel, Functional abdominal pain syndrome, Biliary and Anorectal. The third installment, Rome III was published in 2006, and had some major changes compared to Rome II that came out in 1992 [6]. IBS was divided into different subtypes, including IBS-D (Diarrheal type), IBS-C (constipation type), IBS-M (Mixed type) and IBS-U (Unclassified), and the symptom of bloating was removed from the definition as a primary symptom. In Rome IV, the latest version that was released in May 2016, the term abdominal ‘discomfort’ was removed from the diagnostic criteria of IBS and the frequency of abdominal pain was increased to at least 1 time/ week. Hence, they constitute a more severe subgroup of patients [7].

Out of all the recognizable Functional Gastrointestinal disorders, Functional Dyspepsia is the most common. IBS has a worldwide prevalence in the range of 5–26% [8]. The prevalence of IBS, however, does vary in different regions based on the area, culture and ethnicity, gender and age. In a Japanese population, IBS was the second highest prevalent (9.1%) [8] FBD while in Taiwan and India it was the third most prevalent FBD at 4.4% [9] and 2.7% [10] respectively. The prevalence of IBS is 1.67 times higher in women than in men and lower in patients older than 50 years of age compared to patients younger than 50 years, according to a meta-analysis [11].

Even though there is no increase in mortality associated with FGID, there is a significant rise in morbidity which negatively impacts the quality of life and leads to a greater utilisation of healthcare [12].

To the best of our knowledge, there has been no report on the population-based prevalence of FGID in Pakistan. Information on health status of the FGID subjects in Pakistan is scanty. There is also a dearth of research on community based endoscopic studies and their impact on psychosocial status. One study reported the frequency of IBS to be 14%, with males being affected more than females [13]. Other studies have evaluated the prevalence of IBS amongst healthcare professionals [14] and medical students [15]. Since limited analytical based literature exists regarding FGIDs in Pakistan and other Asian countries, we intend to perform a cross sectional survey to determine the prevalence of FGIDs in a Pakistani population.

The objectives of this study were to determine the prevalence of bowel disorders among adult population living in Karachi-Pakistan using the Rome III functional bowel disorder questionnaire. It also aims to determine the frequency of psychosocial alarm symptoms in patients with bowel disorders, and the degree of overlap between different subtypes of FGIDs.

## Methods

### Study design and setting

This was a community based cross-sectional study conducted in a Sultanabad community, a middle to low-income community, in the city of Karachi, Pakistan. Karachi is the largest city of Pakistan and the capital of Sindh. The study duration was August, 2017 to August, 2019.

### Study population

In the chosen area of the city and its neighbouring community, to represent different socioeconomic strata, multi-stage cluster sampling technique was applied. Households were chosen using systematic sampling technique with random start. A total of 1062 households were initially randomly selected for interviews from the sampled households. Only one person from each household was enrolled in the study. In the presence of two or more eligible persons in a household, balloting was done to select one of them. In cases where no person was present in the sampled house (let suppose in “House A”), we moved to the house very next to it. In case of no response from the next house, the house from the next K^th^ number was selected. For no response houses, we visited each of them at least 2 times before moving on to recruit the next house. Out of 1062 individuals initially selected, 162 individuals dropped out (133 individuals refused to participate in the study, and there was no response from 29 households). Out of the 900 remaining households, we excluded 40 individuals due to the presence of cognitive impairment (10 individuals), severe comorbidities (12 individuals) and alarm symptoms (18 individuals).

### Eligibility

Our study population was living in Karachi and met the following criteria for selection of study population.

- We included adults ≥ 18 years (both genders) from study area of Karachi.
- Those who agreed to give written informed consent to participate.
- We excluded participants who were:
- Mentally disabled or cognitively impaired,
- Complaining of alarm symptoms such as black stools/hematochezia, weight loss, dysphagia, or hematemesis,
- Having severe comorbidities like known cancer and myocardial infarction etc.

(For screening purpose, the mini mental examination was performed to assess cognitive impairment. The purpose of performing cognitive function assessment was that the Rome III adult questionnaire was not valid for cognitive impaired individuals. Those scoring less than 23 (having an educational background) or 21 (no educational background) were labelled to have cognitive impairment and were excluded).

### ROME III questionnaire

A detailed standard ROME III questionnaire for Functional gastrointestinal disorders and the ROME III standard questionnaire for assessing the Psychosocial Alarm symptoms were used to collect information. The standard questionnaires, originally in English language were translated to Urdu, by a professional translator.

The Standard ROME III questionnaire was adapted into Urdu through a process of translation and back translation prior to the study. First, services were acquired from two professional translators who are native speakers of Urdu and also fluent in English, translated the standard ROME III questionnaires independently. Next, a native speaker of English, who was also well versed in Urdu translated the Urdu version back into English. A comparison was made in the original and the back-translated English version to identify any mistranslations or misunderstandings. After carefully rectifying any errors in translation or accuracy, a second Urdu version was prepared and was sent for feedback to 10 gastroenterologists. Before actual data collection, the final translated version of the questionnaire was pre-tested on 45 individuals (5% of the sample size) to assess the flow and clarity of questions. The pre-testing helped data collectors in using the tool comfortably and some of the questions were paraphrased/re-translated in order to get the required response. The interviewers were hired and specially trained to administer the questionnaire to the study subjects. The process of translation of the ROME III form and training of the interviewers was done before the availability of ROME IV, hence we had to go forward with completing our study with ROME III criteria.

The ROME III psychosocial alarm questionnaire consists of seven questions to screen for major distress factors. It covers anxiety (In the last week, have you felt tense or wound up?), depression (In the last week, have you felt downhearted and low?), pain severity (During the last 4‐week, how much bodily pain have you had?), suicidal ideation (Have you felt recently so low that you felt like hurting or killing yourself?), impairment (‘During the last 4‐week, how much did pain/other symptoms interfere with your normal activities?), impaired coping (When I have pain/other symptoms, these appear to be terrible and never get better?) and abuse (Have you been physically, emotionally, sexually victimized any time). For anxiety, responses including *‘most of the time’* and *‘lot of time’* were taken as positive. For depression, responses such as ‘*most of the time’* and *‘a good bit of time’* were considered positive. For suicidal ideation, *‘Often’* and *‘Occasionally’* were considered as positive responses, whereas for pain severity, *‘Very severe’* and *‘Severe’* were positive. In order to assess impairment, *‘Extremely’* and ‘*Quite a bit’* and for impaired coping ‘*Always’* and ‘*Sometime’* were taken as positive responses. The question related to abuse had ‘yes’ and ‘no’ as the answer choices. This rationale for scoring is provided in the questionnaire.

### Recruitment of subjects

Once identified, the participant was introduced to the study, its purpose, details about the objectives, procedures and the possible consequences of the study. After which they were screened for the eligibility. The eligibility was affirmed through a screening log regarding presence of cancer, myocardial infarction and questions related to mini-mental state examination. The subjects were asked to provide a written informed consent to allow us to screen them and for participation if found eligible. Those individuals who were eligible as well as who agreed to participate were recruited.

Participating individuals were requested to fill out a structured interview questionnaire which was administered to them by a trained interviewer. This included information on demographic and household variables, housing and living standards. It also included the ROME III adult questionnaire for identification of FGID as well as the ROME III psychosocial alarm questionnaire. In addition to administering these questionnaires, 10 ml of venous blood was collected for testing Liver function tests (LFTs), TSH, Creatinine and HbA1C, and ultrasound was performed. Participants with abnormal lab tests and/or ultrasound findings were further referred to specialists for management. All those participants who had alarm symptoms, mainly blood in stool or black stools, hematemesis, weight loss, or dysphagia were referred for endoscopy, and subsequently excluded from the study.

### Cognitive impairment screening

Mini-mental state examination questionnaire was used to screen for presence of any cognitive impairment.

### Household information

The first part of questionnaire consisted of information regarding the family structure, total members in the households, the ownership of the house and religion etc.

### Demographic characteristics

This part of the questionnaire included questions regarding the age, gender, education level, occupation, marital status etc.

### Housing and living standards

The information regarding facilities available in the house like the household possessions (television, refrigerator, air conditioner, sewing machine, electric generator, UPS etc.), number of rooms, toilet facility, water and fuel facility were gathered from this section in the questionnaire.

### ROME III adult questionnaire

This was a 93 questions-based tool administered for identifying the FGIDs.

### ROME III psychosocial alarm questionnaire

Before actual data collection, the questionnaire was pre-tested on 45 individuals (5% of the sample size) to assess the flow and clarity of questions. The pre-testing helped data collectors in using the tool comfortably and some of the questions were paraphrased/re-translated in order to get the required response.

### Blood sample collection

In addition to administering questionnaires, blood samples were obtained from a sub-sample of participants. A 10 ml of venous blood was collected and a panel of routing screening blood tests were done to exclude organic pathology.

### Statistical methods

Continuous variables were reported as mean ± SD and frequencies (%) were computed for categorical variables for the characteristics of participating subjects. We assessed statistical differences in proportions between FGIDs Vs. non FGIDs with other covariates using a chi-square test and Fisher exact test, as appropriate. Mean difference proportions between FGIDs Vs. non FGIDs with other covariates using student t-test or Mann-Whitney U test, as appropriate. Sex difference among FGIDs group were compared using Pearson Chi-square test. Mean age was compared between groups using Student’s t-test.

A difference with a p-value of <0.05 was considered statistically significant. All analysis was performed using SPSS version 19.0 and display using Microsoft Excel (office 365).

### Sample size for prevalence of gastrointestinal disorders

There are no published literatures available about the prevalence of complete gastrointestinal disorders of people living in Karachi. However, different studies looking at the frequency and prevalence of individual bowel disorders from Pakistan and other regional countries report the prevalence within a range of 30–45% [16]. This figure was then used to calculate the sample size. With a confidence interval of 95% and bound on error of 5% the sample size came out to be 390.

### Sample size for determining the psychosocial factors with bowel disorders

The proportions of psychosocial factors like anxiety, depression, suicidal ideas, pain severity, impairment and abuse were identified from the literature. The proportions of all these factors varied from 25–60% in the normal adult population of Pakistan. Taking into account these proportions together with 95% confidence interval, 80% power and odd ratio of 2.00 the required sample size came out to be 500. By considering the design effect of 1.7 that occurs for using a multistage cluster sampling, the required sample size came out to be 850.

### Patient and public involvement

There was no involvement of the patients or the public in the design, or conduct, or reporting, or dissemination plans of our research

Ethical approval was obtained from the Ethics research committee of the Aga Khan University. All research was performed in accordance with relevant guidelines/regulations and a written informed consent was obtained from all the participants.

## Results

A total of 860 subjects participated in the present study. FGIDs were diagnosed in 468 (54.4%) patients consisting of 83 (17.7%) males and 385 (82.3%) females. Mean age was higher in participants that met the Rome III symptom-based criteria for FGIDs, compared to those without FGIDs (38.9 ± 11.2 and 35.3 ± 11.4 years, respectively; p<0.001). 47% of participants with FGIDS had no educational background as compared to 31.9% of those without FGIDs, whereas 3.2% of the participants with FGIDS had an education level of at least bachelors or above in comparison to 6.4% of those without FGIDS with same education level. With respect to gender, 49% of the females had no educational background as compared to 13.5% of the males. 20.5% of the males had intermediate level education and 8.4% were educated till bachelor’s or above. Whereas, only 10.2% females were educated till intermediate level and 3.4% had an education of at least bachelors or above. A high proportion of participants that met the Rome III symptom-based criteria for FGIDs were married, whereas very few were either single or widowed/divorced. A similar pattern of marital status was observed amongst participants without FGIDs. Majority of the participants from both the groups, FGIDs and non FGIDS, lived in a nuclear family setup.

Baseline characteristics of patient’s population are shown in **Table 1**.

**Table 1:** Characteristics of study population (n = 860).

	Total	FGIDS; n = 468	No FGIDS; n = 392	*p* value
Age, years	37.2 ± 11.4	38.9 ± 11.2	35.3 ± 11.4	<0.001
Gender				
Male	215(25)	83(17.7)	132(33.7)	<0.001
Female	645(75)	385(82.3)	260(66.3)	
Education				
No educational background	345(40.1)	220(47)	125(31.9)	<0.001
Schooling	365(42.4)	178(38)	187(47.7)	
Intermediate	110(12.8)	55(11.8)	55(14.0)	
Bachelor and above	40(4.7)	15(3.2)	25(6.4)	
Marital status				
Married	707(82.2)	386(82.5)	321(81.9)	0.03
Single	98(11.4)	45(9.6)	53(13.5)	
Divorce/widow	55(6.4)	37(7.9)	18(4.6)	
Family setup				
Live with parents	266(30.9)	130(27.8)	136(34.7)	0.02
Nuclear family	594(69.1)	338(72.2)	256(65.3)	
Ethnicity				
Sindhi	19(2.2)	9(1.9)	10(2.6)	0.40
Punjabi	38(4.4)	16(3.4)	22(5.6)	
Pakhtun	288(33.5)	166(35.5)	122(31.1)	
Balochi	2(0.2)	1(0.2)	1(0.3)	
Others	513(59.7)	276(59)	237(60.5)	

*Mann-Whitney U test

### Spectrum of gastrointestinal disorders

In all participants with the functional disorders, frequency of the esophageal, gastroduodenal, bowel, abdominal pain syndrome and anorectal disorders was 34.0%, 38.3%, 43.4%, 15.8% and 1.0%, respectively. Overall, the most prevalent FGID was Functional dyspepsia, followed by functional heartburn and bloating.

In the functional esophageal disorders, functional heartburn was observed the most frequently. In functional gastroduodenal disorders, FD was the most prevalent, which include both postprandial Distress syndrome and Epigastric pain syndrome. In Functional Bowel Disorders, Functional Bloating was the most prevalent, followed by Unspecified Functional Bowel Disorder.

Only 74 (8.6%) participants had Irritable Bowel syndrome. In Functional Anorectal Disorders, Functional Fecal Incontinence was the most prevalent followed by Functional Anorectal Pain. There weren’t any participants with Functional Defecation Disorder. **Table 2** shows the detailed spectrum.

**Table 2:** Functional Gastrointestinal disorders (n = 860).

	Total N = 860	Male; n = 215	Female; n = 645	*p* value
**Functional Gastrointestinal Disorders (FGIDS)**	468(54.5)	83(38.6)	385(59.7)	<0.001
**Functional esophageal disorders (FES)**	**292(34)**	**43(20)**	**249(38.6)**	<0.001
Heartburn	276(32.1)	42(19.5)	234(36.3)	<0.001
Chest pain of Presumed Esophageal Origin	3(0.3)	0	3(0.5)	0.57
Dysphagia	5(0.6)	0	5(0.8)	0.33
Globus	10(1.2)	1(0.5)	9(1.4)	0.46
**Functional Gastroduodenal Disorders (FGD)**	**329(38.3)**	**47(21.9)**	**282(43.7)**	<0.001
Functional Dyspepsia	329(38.3)	47(21.9)	282(43.7)	<0.001
Postprandial Distress Syndrome	95(11.0)	16(7.4)	79(12.2)	0.052
Epigastric Pain syndrome	28(3.2)	7(3.3)	21(3.3)	0.99
PPD and EPS Overlap	6(0.6)	4(1.8)	2(0.31)	0.03
**Functional Bowel Disorders (FBD)**	**373(43.4)**	**59(27.4)**	**314(48.7)**	<0.001
Irritable Bowel Syndrome	74(8.6)	4(1.9)	70(10.9)	<0.001
Functional Bloating	265(30.8)	34(15.8)	231(35.8)	<0.001
Functional Diarrhea	1(0.1)	1(0.5)	0	0.25
Unspecified Functional Bowel Disorder	127(14.8)	30(14)	97(15)	0.69
**Functional abdominal pain syndrome (FAPS)**	**136(15.8)**	**12(5.6)**	**124(19.2)**	<0.001
**Functional Anorectal Disorder (FAD**	**9(1.0)**	**2(0.9)**	**7(1.1)**	0.99
Functional Fecal Incontinence	9(1.0)	2(0.9)	7(1.1)	0.99

**Fig 1:**
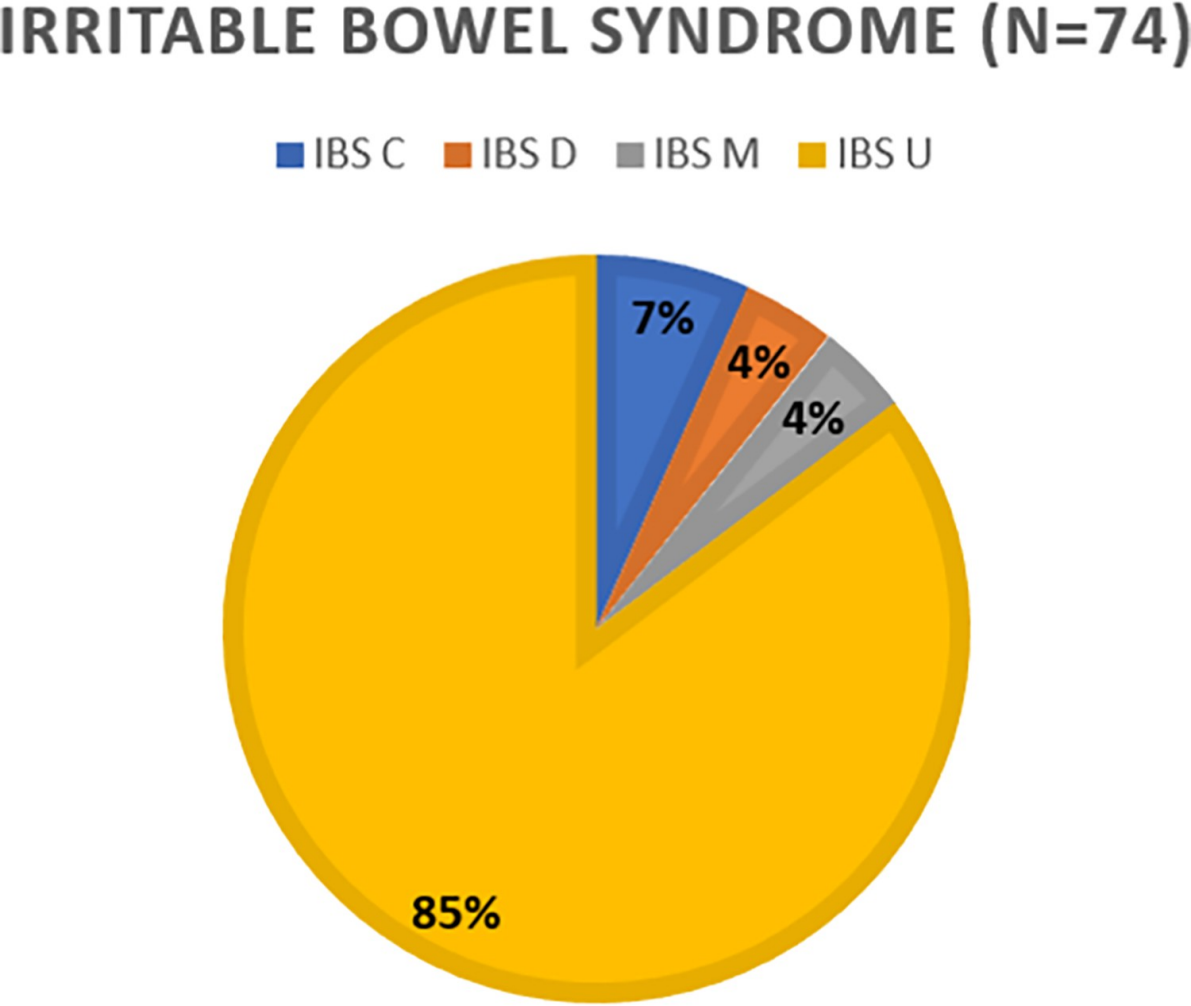
Sub classification of irritable bowel syndrome.

As illustrated in **Fig 1**, out of 78 participants who met the criteria for Irritable Bowel syndrome, majority had IBS-Unspecified (86.5%), followed by IBS-C (6.8%), IBS-D (4.1%) and IBS-M (4.1%).

### Overlap of Functional Gastrointestinal disorders

**Fig 2:**
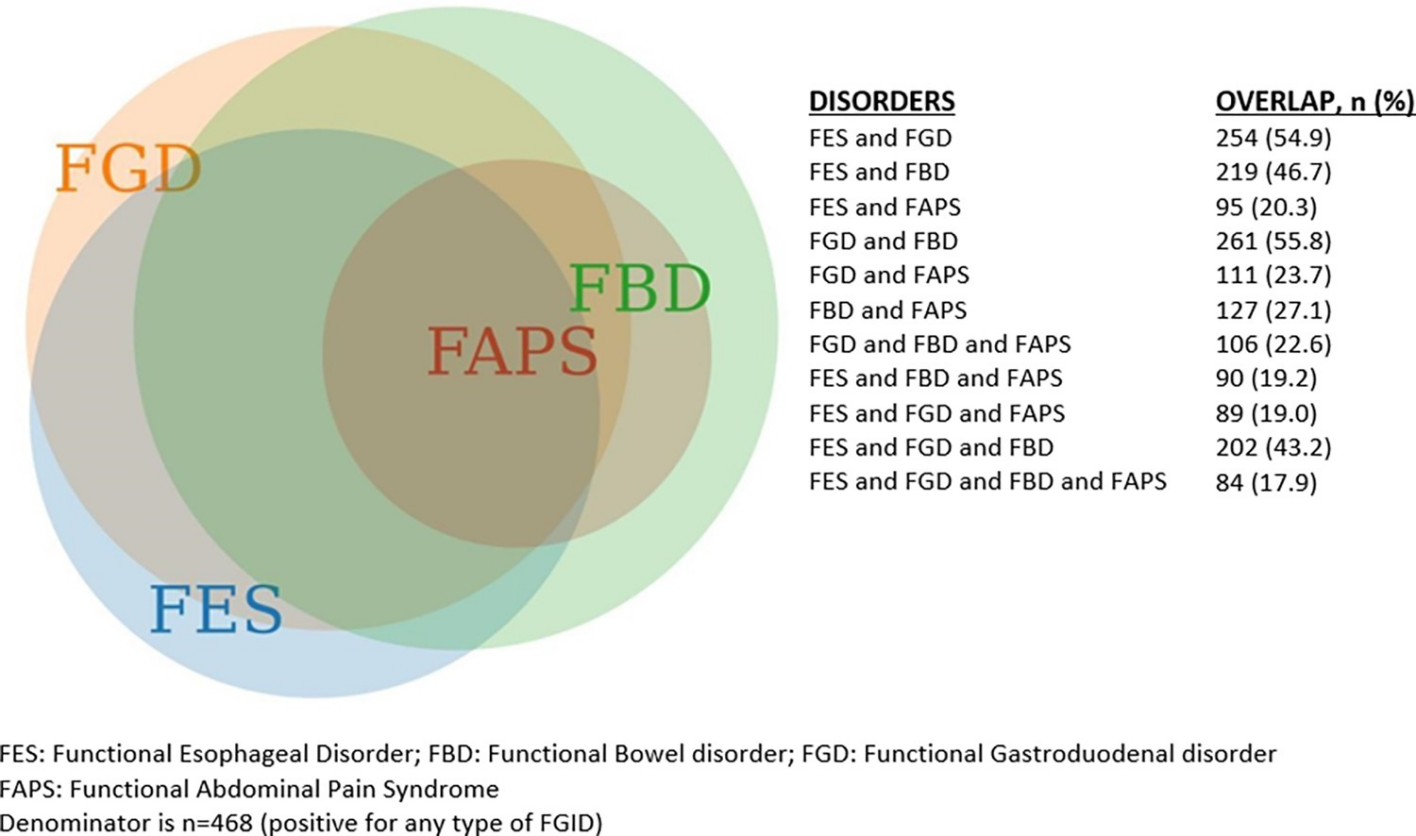
Overlap between Functional Gastrointestinal disorders.

Among a total of 468 participants with FGIDS, 347 (74.1%) had overlapping FGID, while only 121 (25.8%) had an isolated FGID. Out of those with non overlapping FGID, 80 participants had only FBD while only 4 had an isolated functional abdominal pain syndrome. 16 and 21 participants had isolated FGD and FES syndromes respectively. The maximum overlap is between Functional bowel disorder and functional gastroduodenal disorder, followed by the overlap between Functional esophageal and functional gastroduodenal disorders. An overlap between all the four major categories of FGIDS were observed in 84 participants. This is illustrated in **Fig 2.**

### Psychosocial symptoms

**Fig 3:**
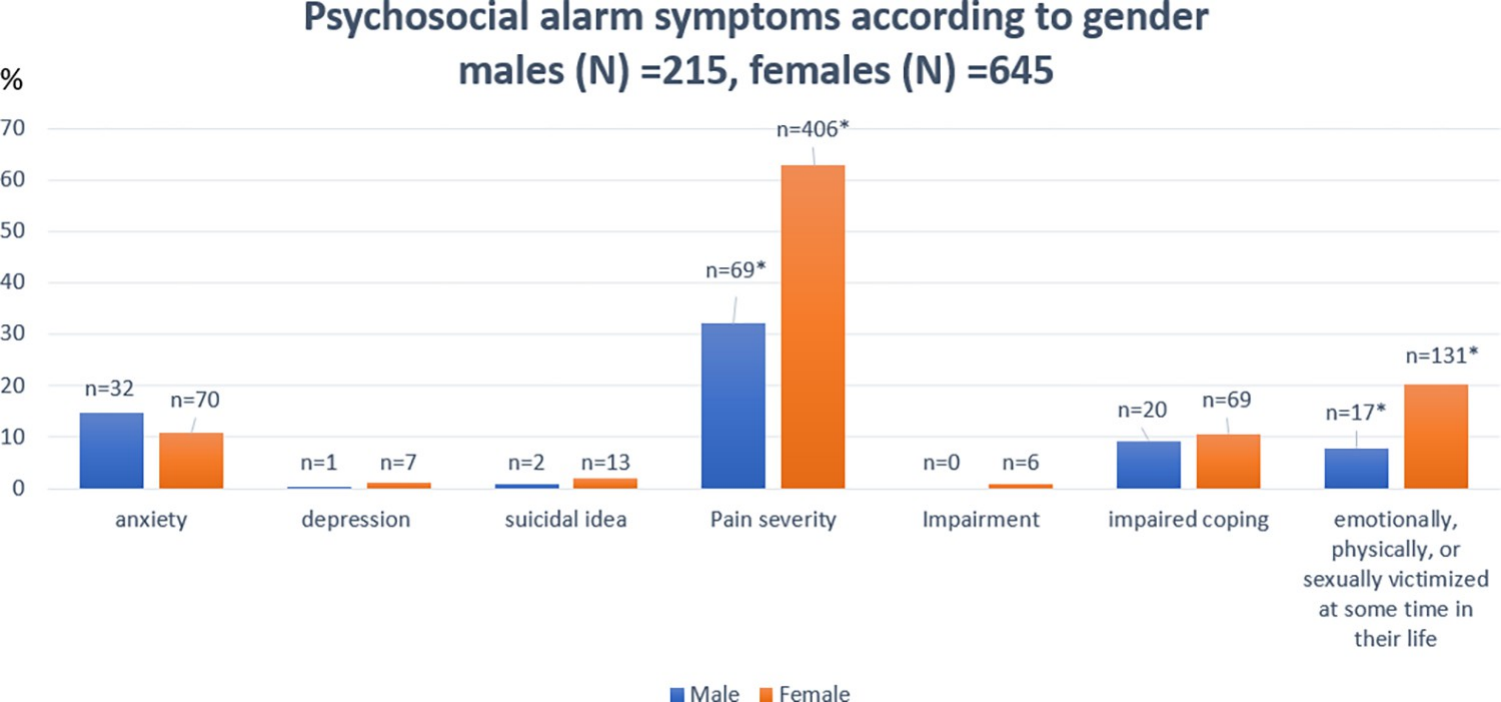
Psychosocial alarm symptoms according to gender (* represents statistically significant).

**Fig 4:**
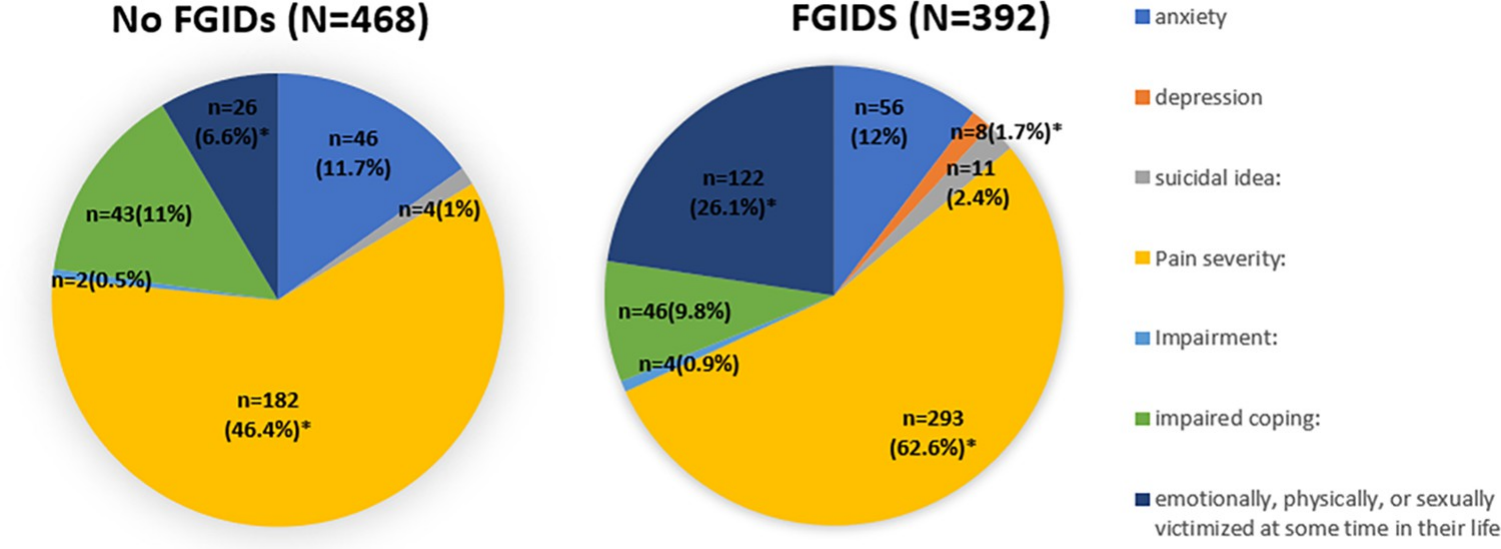
Psychosocial alarms symptoms according to FGID status (* represents statistically significant).

Anxiety was found to be approximately equally present in participants irrespective of their FGID status. However, depression was present in 8 participants, all of whom had some type of a functional GI disorder (p<0.009). The results were statistically significant for 122 participants with FGIDS having gone through emotional, physical, or sexual victimization at some time in their life, whereas only 26 participants who did not have any FGIDs also reported the same (p<0.001). More participants with FGIDs (62.6%) also reported a higher pain severity compared to patents without FGIDS (46.4%), p value <0/001. With respect to gender, a higher proportion of females compared to males reported higher pain severity (62.9% vs 32.1%, p<0.001) and feeling of emotionally, sexually, and physically victimized at some time in their life. (20.3% vs 7.9% p<0.001) The distribution of these psychosomatic symptoms in relation to the participants and gender and FGID status is provided in **Figs 3 and 4** respectively.

## Discussion

To the best of our knowledge, this is the first community-based study to estimate the spectra, psychosocial alarm symptoms and the overlap amongst all FGIDs in Pakistan using the Rome III integrated questionnaire and scoring algorithm. Overall, our study provides a detailed spectrum, of 13 disorders, diagnosed amongst 860 patients. There is also a possibility of coexistence of multiple functional disorders in the same patient, according to the ROME III criteria and we have highlighted that in our study.

### General demographics

Overall, we had a higher proportion of female participants compared to male participants in the study, likely because the community-based survey was administered during daytime and nonworking females were mostly present in their homes. Also, the proportion of females compared to males who screened positive for at least one type of FGID was significantly higher. A higher proportion of male participants compared to female participants had an education of intermediate level (12^th^ grade or high school), bachelors or above. Whereas, almost half of the female participants in our study had no educational background. This highlights the gender discrimination in education, which mainly exists in communities with low socio-economic statuses.

### Spectrum of FGIDS

In this study, approximately half of the participants had some type of functional gastrointestinal disorder. Since this is the first large scale study conducted in our population, no comparison can be made locally. A global study conducted across 33 countries through internet surveys reported a worldwide prevalence of 40.3%, much lower than our rate [17]. Our prevalence rate of FGID is comparable with China (55.7%) [18], Japan outpatients (57.4%) [19], and tertiary hospitals in Korea (53.5%) [20] but much higher compared to Taiwan (26.2%) [9]. The most prevalent domain in our study was FBD. The prevalence rate of FBD is consistent with studies conducted in Japan (45.6%) [8] and China (41.6%) [21], but is significantly higher compared to Taiwan (26.2%) [9] and Iran (10.9%) [22] Since the prevalence of all the individual domains of FGIDs have been investigated in only a handful of studies, including one in South China [23] and Korea [20], most of the prevalence of FGID recorded worldwide are underestimates. The differences in the prevalence of FGIDs exist due to cultural, economical and diet related disparities amongst different geographical regions.

In our population, Functional Dyspepsia was followed by functional heartburn and functional bloating. The diagnosis of functional heartburn requires oesophageal pH studies as well as endoscopy, to rule out erosive and non erosive esophagitis. Since endoscopies were not performed, heartburn of a purely functional origin was not differentiated from the erosive and non erosive types of heartburn in our study. Hence, such a high prevalence of functional heartburn could be an overestimate. Surprisingly, we did not find any patient who fulfilled the criteria for Functional Constipation despite it being a common disorder world wide.

Amongst all the participants diagnosed with functional dyspepsia, we had a relatively small proportion of people who fulfilled the criteria for PPD and EPS. This could be a result of misinterpretation of the sub-categories in the FD questionnaire by the study participants. However, it does not undermine the fact that a very high proportion of our population is suffering from Functional Dyspepsia. In addition, similar to our findings, most of the studies conducted using the ROME III criteria in different populations of East Asia such as in Bangladesh [10], Korea [20] and Japan [24], reported the prevalence of PPD to be higher than EPS. In a Korean study conducted by Park et al., there were relatively fewer patients with neither PPD nor EPS (17.6%) but a similar result on overlap syndrome of both PPD and EPS (2.2%) [20].

Despite being considered as one of most common FGIDs worldwide previously, recent studies have been reporting a relatively low prevalence of IBS in recent times. In a worldwide study, 19 out of 26 countries reported the rates of IBS to be between 3%-5% [17]. Similarly, we found its prevalence in our population to be lower down in the list of FGIDs. The prevalence of IBS in our study population is similar to the reports of other east Asian countries such as China (10.4% and 15.9%) [25], Taiwan (4.4%) [9] and Korea (9%) [26]. IBS was the second most common type of FGID in South China and Korea, after FD [20, 23]. In Japan the prevalence ranged between 1.1 to 29.2% [27] whereas in a systematic review of IBS in North America the range of IBS was found to be between 3 to 20% with the highest estimates between 10 to 15% [28]. In contrast, Sorouri et al. reported a very low prevalence of IBS (1.1%) in an Iranian population, one of the lowest prevalence recorded worldwide [22]. Recent studies using the Rome IV diagnostic criteria for IBS report a much lower prevalence rate of IBS compared to the ones using ROME III, or older versions of the ROME criteria, probably due to a higher minimum pain frequency in ROME IV, as reported by a study conducted across United States, United Kingdom and Canada [29].

### Subtypes of IBS

The questionnaire-based classification of the subtypes of IBS is determined by the stool frequency and consistency. The major IBS subtype in our study was IBS-U, followed by IBS-C, and then IBS-M and IBS-D. This is quite significant and different from the data compiled from other Asian countries. Out of the 8 studies that reported the prevalence of IBS based on Rome III diagnostic criteria, 5 reported the proportion of IBS-U to be between 12.1–32.7% [25]. The same review on Asian countries reported the proportion of Diarrhoea dominant IBS to be between 0.8% to 74%, while that of constipation dominant IBS to be between 12% to 77% [25]. Generally, IBS- diarrhoea is the predominant subtype of Asia whereas IBS-C is the predominant subtype of Europe, owing to the presumption that low fibre diet is common in the west [18]. There is a high variability in the reports of IBS subtypes, perhaps because this is a very subjective criteria for both the physician and the patients. Precise interpretation of abdominal pain or discomfort along with the stool characteristics is required. There is also a lack of agreement on a standard diagnostic criterion for sub-classification of IBS.

### Overlap

According to the Rome III criteria, there can be multiple FGID coexistent in one person. Our result on overlapping FGIDs is slightly/ somewhat higher compared to data from South China (50.3%) [23], Japan (56.4%) [19] and Korea (51%) [20]. In comparison to Rome II, there is a higher degree of overlap between FGID diagnosed using Rome III [23]. In our study, the maximum overlap was between FD (FGD) and FBD, closely followed by FD and FES. There were only 16 patients who had functional dyspepsia alone, which shows that isolated FD is relatively rare. Most of the studies have reported maximum overlap between FD and IBS. Perveen et al. concluded that amongst patients with IBS, the prevalence of FD varies between 29–97%, while amongst patients with FD, the prevalence of IBS varies between 13–29% [10]. Since the diagnosis of these disorders is based on patients symptoms and interpretation of the questionnaire, there is a possibility of an overdiagnosis of the overlap. However, this does not undermine the fact that a high degree of overlap between disorders exists and makes it necessary for a thorough further investigation to diagnose other types of functional disorders in a patient with at least one diagnosed FGID. It also creates difficulties for management strategies because the patients might not be satisfied with the treatment of one disorder. An example of this is that there is a different response to treatment with acid suppressive therapy in patients with FD and heartburn compared to those with no heartburn [20]. There is a possibility that overlapping could be a result of a common underlying pathophysiological mechanism for functional disorders and the question arises that whether these overlapping disorders should be treated as a single disorder or as multiple disorders [23].

### Psychosocial alarm symptoms

Having a functional disorder can impact one’s mental health and this was evident by the finding that a higher proportion of patients with any FGID were diagnosed with depression, whereas there was no participant without FGID to screen positive for depression. Lee et al. reported that emotional stress and depression were independent risk factors for FD and IBS [30], and Pinto Sanchez et al. reported that there was a proportional increase in depression and anxiety with greater number of FGIDs within the FGID group [31]. It is however difficult to form a causal relationship between anxiety, depression and functional disorders. People with FGIDs could experience both anxiety and/or depression as a result of their chronic illness. Alternatively, patient with underlying depression and/or anxiety could experience functional disorders, where an underlying mental illness serves as a risk factor for FGID. Besides depression, a higher number of participants with FGID also reported a higher pain severity and a feeling of emotionally, physically or sexually victimized. Drossman et al. reported that compared to organic disorders, patients with functional bowel disorders were found to have significantly more experiences of both sexual and physical abuse [32].

There are very few studies that have reported a comprehensive data on all the major categories of the functional gastrointestinal disorder. Most of the studies report the prevalence of functional Bowel disorders which is just one subtype of FGID. This is one of the major strengths of our study.

One limitation of this study is that it is based on the ROME III criteria, and a newer version known as the ROME IV criteria has also come out. Since this study was planned quite earlier and translation of ROME III questionnaire and training of interviewers and pretesting were already done so we continued our study with ROME III questionnaire. In retrospect, we believe that using ROME III was beneficial since most of the prevalence studies are based on ROME III, making our results more comparable to the published literature. Furthermore, a recent systematic review concluded that ‘’prevalence of IBS was substantially lower with the Rome IV criteria, suggesting that these more restrictive criteria might be less suitable than Rome III for population-based epidemiological surveys. This finding suggests that Rome IV appears to be less sensitive compared to Rome III especially in Asian populations [33, 34]. Another limitation is that we had an uneven gender distribution (male 17.7% vs female 82.3%). Since this community-based study was conducted during the day hours, men were out at work and the non-working females were mostly present at home. A possible additional limitation will be that being a population-based study, an esophageoduodenoscopy or colonoscopy was not performed on the participants. Despite the lack of evidence of a structural disease, we would expect the investigations to be negative or normal as others have confirmed [35].

In conclusion this is the first epidemiological study documenting the prevalence of functional disorders from this part of Asia. There is a very high burden of Functional Gastrointestinal disorders, with around half of the population suffering with at least one type of a functional disorder, and a high degree of overlapping FGIDs. This information reiterates the need of further recourse allocation, both on the hospital level and policy making level, for better management of patients with functional disorders.

## Supporting information

S1 File (DOC)

S2 File (DOC)
