# Supplementary material for: Epidemiology of functional gastrointestinal disorders using ROME III adult questionnaire, a population based cross sectional study in Karachi—Pakistan
Source: PLoS One. 2022 Jun 13;17(6):e0268403. doi: 10.1371/journal.pone.0268403 (PMC9191742; doi:10.1371/journal.pone.0268403)
Supplement: S1 File — (DOC) [file pone.0268403.s001.doc]

GET
  FILE='C:\Backup data\Dr Shahab Abid\IBS\data350.sav'.
DATASET NAME DataSet1 WINDOW=FRONT.
FREQUENCIES VARIABLES=C1_IBS
  /ORDER=ANALYSIS.


Frequencies


Notes	
Output Created	24-Jan-2022 15:57:14	
Comments	 	
Input	Data	C:\Backup data\Dr Shahab Abid\IBS\data350.sav	
	Active Dataset	DataSet1	
	Filter	<none>	
	Weight	<none>	
	Split File	<none>	
	N of Rows in Working Data File	857	
Missing Value Handling	Definition of Missing	User-defined missing values are treated as missing.	
	Cases Used	Statistics are based on all cases with valid data.	
Syntax	FREQUENCIES VARIABLES=C1_IBS
  /ORDER=ANALYSIS.
	
Resources	Processor Time	00 00:00:00.015	
	Elapsed Time	00 00:00:00.016	


[DataSet1] C:\Backup data\Dr Shahab Abid\IBS\data350.sav


Statistics	
Irritable Bowel Syndrome	
N	Valid	821	
	Missing	36	


Irritable Bowel Syndrome	
	Frequency	Percent	Valid Percent	Cumulative Percent	
Valid	No	746	87.0	90.9	90.9	
	Yes	75	8.8	9.1	100.0	
	Total	821	95.8	100.0		
Missing	System	36	4.2			
Total	857	100.0			

USE ALL.
COMPUTE filter_$=(C1_IBS=1).
VARIABLE LABELS filter_$ 'C1_IBS=1 (FILTER)'.
VALUE LABELS filter_$ 0 'Not Selected' 1 'Selected'.
FORMATS filter_$ (f1.0).
FILTER BY filter_$.
EXECUTE.
FREQUENCIES VARIABLES=IBS_C IBS_D IBS_M IBS_U
  /ORDER=ANALYSIS.


Frequencies


Notes	
Output Created	24-Jan-2022 15:58:09	
Comments	 	
Input	Data	C:\Backup data\Dr Shahab Abid\IBS\data350.sav	
	Active Dataset	DataSet1	
	Filter	C1_IBS=1 (FILTER)	
	Weight	<none>	
	Split File	<none>	
	N of Rows in Working Data File	75	
Missing Value Handling	Definition of Missing	User-defined missing values are treated as missing.	
	Cases Used	Statistics are based on all cases with valid data.	
Syntax	FREQUENCIES VARIABLES=IBS_C IBS_D IBS_M IBS_U
  /ORDER=ANALYSIS.
	
Resources	Processor Time	00 00:00:00.000	
	Elapsed Time	00 00:00:00.000	


[DataSet1] C:\Backup data\Dr Shahab Abid\IBS\data350.sav


Statistics	
	IBS_C	IBS_D	IBS_M	IBS_U	
N	Valid	75	75	75	75	
	Missing	0	0	0	0	


Frequency Table


IBS_C	
	Frequency	Percent	Valid Percent	Cumulative Percent	
Valid	No	69	92.0	92.0	92.0	
	Yes	6	8.0	8.0	100.0	
	Total	75	100.0	100.0		


IBS_D	
	Frequency	Percent	Valid Percent	Cumulative Percent	
Valid	No	71	94.7	94.7	94.7	
	Yes	4	5.3	5.3	100.0	
	Total	75	100.0	100.0		


IBS_M	
	Frequency	Percent	Valid Percent	Cumulative Percent	
Valid	No	71	94.7	94.7	94.7	
	Yes	4	5.3	5.3	100.0	
	Total	75	100.0	100.0		


IBS_U	
	Frequency	Percent	Valid Percent	Cumulative Percent	
Valid	No	10	13.3	13.3	13.3	
	Yes	65	86.7	86.7	100.0	
	Total	75	100.0	100.0		

FREQUENCIES VARIABLES=B1F_dyspepsis
  /ORDER=ANALYSIS.


Frequencies


Notes	
Output Created	24-Jan-2022 16:00:23	
Comments	 	
Input	Data	C:\Backup data\Dr Shahab Abid\IBS\data350.sav	
	Active Dataset	DataSet1	
	Filter	<none>	
	Weight	<none>	
	Split File	<none>	
	N of Rows in Working Data File	857	
Missing Value Handling	Definition of Missing	User-defined missing values are treated as missing.	
	Cases Used	Statistics are based on all cases with valid data.	
Syntax	FREQUENCIES VARIABLES=B1F_dyspepsis
  /ORDER=ANALYSIS.
	
Resources	Processor Time	00 00:00:00.000	
	Elapsed Time	00 00:00:00.000	


[DataSet1] C:\Backup data\Dr Shahab Abid\IBS\data350.sav


Statistics	
Functional Dyspepsia	
N	Valid	821	
	Missing	36	


Functional Dyspepsia	
	Frequency	Percent	Valid Percent	Cumulative Percent	
Valid	No	494	57.6	60.2	60.2	
	Yes	327	38.2	39.8	100.0	
	Total	821	95.8	100.0		
Missing	System	36	4.2			
Total	857	100.0			
