# Supplementary material for: Epidemiology of functional gastrointestinal disorders using ROME III adult questionnaire, a population based cross sectional study in Karachi—Pakistan
Source: PLoS One. 2022 Jun 13;17(6):e0268403. doi: 10.1371/journal.pone.0268403 (PMC9191742; doi:10.1371/journal.pone.0268403)
Supplement: S2 File — (DOC) [file pone.0268403.s002.doc]

Frequencies

Statistics	
FGIDS	
N	Valid	860	
	Missing	0	


FGIDS	
	Frequency	Percent	Valid Percent	Cumulative Percent	
Valid	No	392	45.6	45.6	45.6	
	Yes	468	54.4	54.4	100.0	
	Total	860	100.0	100.0		

T-Test


Group Statistics	
	FGIDS	N	Mean	Std. Deviation	Std. Error Mean	
 age	Yes	468	38.91	11.208	.518	
	No	392	35.30	11.415	.577	


Independent Samples Test	
	Levene's Test for Equality of Variances	
	F	Sig.	
 age	Equal variances assumed	.265	.607	
	Equal variances not assumed			

Independent Samples Test	
	t-test for Equality of Means	
	t	df	Sig. (2-tailed)	Mean Difference	
 age	Equal variances assumed	4.663	858	.000	3.609	
	Equal variances not assumed	4.656	826.296	.000	3.609	

Independent Samples Test	
	t-test for Equality of Means	
	Std. Error Difference	95% Confidence Interval of the Difference	
		Lower	Upper	
 age	Equal variances assumed	.774	2.090	5.128	
	Equal variances not assumed	.775	2.087	5.130	


Crosstabs


edu_cat * FGIDS
Crosstab	
	FGIDS	Total	
	No	Yes		
edu_cat	no education	Count	125	220	345	
		% within FGIDS	31.9%	47.0%	40.1%	
	schooling	Count	187	178	365	
		% within FGIDS	47.7%	38.0%	42.4%	
	Intermediate	Count	55	55	110	
		% within FGIDS	14.0%	11.8%	12.8%	
	Bachelor and above	Count	25	15	40	
		% within FGIDS	6.4%	3.2%	4.7%	
Total	Count	392	468	860	
	% within FGIDS	100.0%	100.0%	100.0%	


Chi-Square Tests	
	Value	df	Asymp. Sig. (2-sided)	
Pearson Chi-Square	22.340a	3	.000	
Likelihood Ratio	22.524	3	.000	
Linear-by-Linear Association	17.598	1	.000	
N of Valid Cases	860			
a. 0 cells (.0%) have expected count less than 5. The minimum expected count is 18.23.
	


mstatus * FGIDS
Crosstab	
	FGIDS	Total	
	No	Yes		
mstatus	married	Count	321	386	707	
		% within FGIDS	81.9%	82.5%	82.2%	
	single	Count	53	45	98	
		% within FGIDS	13.5%	9.6%	11.4%	
	divorced/widow	Count	18	37	55	
		% within FGIDS	4.6%	7.9%	6.4%	
Total	Count	392	468	860	
	% within FGIDS	100.0%	100.0%	100.0%	


Chi-Square Tests	
	Value	df	Asymp. Sig. (2-sided)	
Pearson Chi-Square	6.527a	2	.038	
Likelihood Ratio	6.614	2	.037	
Linear-by-Linear Association	.508	1	.476	
N of Valid Cases	860			
a. 0 cells (.0%) have expected count less than 5. The minimum expected count is 25.07.
	


Family setup * FGIDS
Crosstab	
	FGIDS	Total	
	No	Yes		
Family setup	live with parents	Count	136	130	266	
		% within FGIDS	34.7%	27.8%	30.9%	
	single family	Count	256	338	594	
		% within FGIDS	65.3%	72.2%	69.1%	
Total	Count	392	468	860	
	% within FGIDS	100.0%	100.0%	100.0%	


Chi-Square Tests	
	Value	df	Asymp. Sig. (2-sided)	Exact Sig. (2-sided)	Exact Sig. (1-sided)	
Pearson Chi-Square	4.776a	1	.029			
Continuity Correctionb	4.458	1	.035			
Likelihood Ratio	4.766	1	.029			
Fisher's Exact Test				.032	.017	
Linear-by-Linear Association	4.771	1	.029			
N of Valid Cases	860					
a. 0 cells (.0%) have expected count less than 5. The minimum expected count is 121.25.
b. Computed only for a 2x2 table
	
 Ethnicity * FGIDS
Crosstab	
	FGIDS	Total	
	No	Yes		
 Ethnicity	sindhi	Count	10	9	19	
		% within FGIDS	2.6%	1.9%	2.2%	
	punjabi	Count	22	16	38	
		% within FGIDS	5.6%	3.4%	4.4%	
	pakhtun	Count	122	166	288	
		% within FGIDS	31.1%	35.5%	33.5%	
	Balochi	Count	1	1	2	
		% within FGIDS	.3%	.2%	.2%	
	others	Count	237	276	513	
		% within FGIDS	60.5%	59.0%	59.7%	
Total	Count	392	468	860	
	% within FGIDS	100.0%	100.0%	100.0%	


Chi-Square Tests	
	Value	df	Asymp. Sig. (2-sided)	
Pearson Chi-Square	4.002a	4	.406	
Likelihood Ratio	3.995	4	.407	
Linear-by-Linear Association	.009	1	.925	
N of Valid Cases	860			
a. 2 cells (20.0%) have expected count less than 5. The minimum expected count is .91.
	


Crosstabs

 gender * FGIDS Crosstabulation	
	FGIDS	Total	
	No	Yes		
 gender	Male	Count	132	83	215	
		% within FGIDS	33.7%	17.7%	25.0%	
	female	Count	260	385	645	
		% within FGIDS	66.3%	82.3%	75.0%	
Total	Count	392	468	860	
	% within FGIDS	100.0%	100.0%	100.0%	


Chi-Square Tests	
	Value	df	Asymp. Sig. (2-sided)	Exact Sig. (2-sided)	Exact Sig. (1-sided)	
Pearson Chi-Square	28.902a	1	.000			
Continuity Correctionb	28.058	1	.000			
Likelihood Ratio	28.920	1	.000			
Fisher's Exact Test				.000	.000	
Linear-by-Linear Association	28.868	1	.000			
N of Valid Cases	860					
a. 0 cells (.0%) have expected count less than 5. The minimum expected count is 98.00.
b. Computed only for a 2x2 table
	

FGIDS *  gender Crosstabulation	
	 gender	Total	
	Male	female		
FGIDS	No	Count	132	260	392	
		% within  gender	61.4%	40.3%	45.6%	
	Yes	Count	83	385	468	
		% within  gender	38.6%	59.7%	54.4%	
Total	Count	215	645	860	
	% within  gender	100.0%	100.0%	100.0%	


Chi-Square Tests	
	Value	df	Asymp. Sig. (2-sided)	Exact Sig. (2-sided)	Exact Sig. (1-sided)	
Pearson Chi-Square	28.902a	1	.000			
Continuity Correctionb	28.058	1	.000			
Likelihood Ratio	28.920	1	.000			
Fisher's Exact Test				.000	.000	
Linear-by-Linear Association	28.868	1	.000			
N of Valid Cases	860					
a. 0 cells (.0%) have expected count less than 5. The minimum expected count is 98.00.
b. Computed only for a 2x2 table
	

CROSSTABS
  /TABLES=FED A1_FHeartburn A2_FCpain A3_FDysphagia A4_Globus BY GENDER
  /FORMAT=AVALUE TABLES
  /STATISTICS=CHISQ
  /CELLS=COUNT COLUMN
  /COUNT ROUND CELL.

F Esophageal disorder *  gender
Crosstab	
	 gender	Total	
	Male	female		
F Esophageal disorder	No	Count	172	396	568	
		% within  gender	80.0%	61.4%	66.0%	
	Yes	Count	43	249	292	
		% within  gender	20.0%	38.6%	34.0%	
Total	Count	215	645	860	
	% within  gender	100.0%	100.0%	100.0%	


Chi-Square Tests	
	Value	df	Asymp. Sig. (2-sided)	Exact Sig. (2-sided)	Exact Sig. (1-sided)	
Pearson Chi-Square	24.889a	1	.000			
Continuity Correctionb	24.066	1	.000			
Likelihood Ratio	26.515	1	.000			
Fisher's Exact Test				.000	.000	
Linear-by-Linear Association	24.860	1	.000			
N of Valid Cases	860					
a. 0 cells (.0%) have expected count less than 5. The minimum expected count is 73.00.
b. Computed only for a 2x2 table
	

functional heart burn *  gender
Crosstab	
	 gender	Total	
	Male	female		
functional heart burn	No	Count	173	411	584	
		% within  gender	80.5%	63.7%	67.9%	
	Yes	Count	42	234	276	
		% within  gender	19.5%	36.3%	32.1%	
Total	Count	215	645	860	
	% within  gender	100.0%	100.0%	100.0%	


Chi-Square Tests	
	Value	df	Asymp. Sig. (2-sided)	Exact Sig. (2-sided)	Exact Sig. (1-sided)	
Pearson Chi-Square	20.744a	1	.000			
Continuity Correctionb	19.983	1	.000			
Likelihood Ratio	22.088	1	.000			
Fisher's Exact Test				.000	.000	
Linear-by-Linear Association	20.720	1	.000			
N of Valid Cases	860					
a. 0 cells (.0%) have expected count less than 5. The minimum expected count is 69.00.
b. Computed only for a 2x2 table
	


functional chest pain *  gender
Crosstab	
	 gender	Total	
	Male	female		
functional chest pain	No	Count	215	642	857	
		% within  gender	100.0%	99.5%	99.7%	
	Yes	Count	0	3	3	
		% within  gender	.0%	.5%	.3%	
Total	Count	215	645	860	
	% within  gender	100.0%	100.0%	100.0%	


Chi-Square Tests	
	Value	df	Asymp. Sig. (2-sided)	Exact Sig. (2-sided)	Exact Sig. (1-sided)	
Pearson Chi-Square	1.004a	1	.316			
Continuity Correctionb	.112	1	.738			
Likelihood Ratio	1.730	1	.188			
Fisher's Exact Test				.577	.421	
Linear-by-Linear Association	1.002	1	.317			
N of Valid Cases	860					
a. 2 cells (50.0%) have expected count less than 5. The minimum expected count is .75.
b. Computed only for a 2x2 table
	

Functional Dysphagia *  gender
Crosstab	
	 gender	Total	
	Male	female		
Functional Dysphagia	No	Count	215	640	855	
		% within  gender	100.0%	99.2%	99.4%	
	Yes	Count	0	5	5	
		% within  gender	.0%	.8%	.6%	
Total	Count	215	645	860	
	% within  gender	100.0%	100.0%	100.0%	


Chi-Square Tests	
	Value	df	Asymp. Sig. (2-sided)	Exact Sig. (2-sided)	Exact Sig. (1-sided)	
Pearson Chi-Square	1.676a	1	.195			
Continuity Correctionb	.604	1	.437			
Likelihood Ratio	2.887	1	.089			
Fisher's Exact Test				.339	.236	
Linear-by-Linear Association	1.674	1	.196			
N of Valid Cases	860					
a. 2 cells (50.0%) have expected count less than 5. The minimum expected count is 1.25.
b. Computed only for a 2x2 table
	

A4_Globus *  gender
Crosstab	
	 gender	Total	
	Male	female		
A4_Globus	No	Count	214	636	850	
		% within  gender	99.5%	98.6%	98.8%	
	Yes	Count	1	9	10	
		% within  gender	.5%	1.4%	1.2%	
Total	Count	215	645	860	
	% within  gender	100.0%	100.0%	100.0%	


Chi-Square Tests	
	Value	df	Asymp. Sig. (2-sided)	Exact Sig. (2-sided)	Exact Sig. (1-sided)	
Pearson Chi-Square	1.214a	1	.271			
Continuity Correctionb	.540	1	.463			
Likelihood Ratio	1.463	1	.226			
Fisher's Exact Test				.465	.242	
Linear-by-Linear Association	1.213	1	.271			
N of Valid Cases	860					
a. 1 cells (25.0%) have expected count less than 5. The minimum expected count is 2.50.
b. Computed only for a 2x2 table
	

F. Gastroduodenal disorders *  gender
Crosstab	
	 gender	Total	
	Male	female		
F. Gastroduodenal disorders	No	Count	168	363	531	
		% within  gender	78.1%	56.3%	61.7%	
	Yes	Count	47	282	329	
		% within  gender	21.9%	43.7%	38.3%	
Total	Count	215	645	860	
	% within  gender	100.0%	100.0%	100.0%	


Chi-Square Tests	
	Value	df	Asymp. Sig. (2-sided)	Exact Sig. (2-sided)	Exact Sig. (1-sided)	
Pearson Chi-Square	32.623a	1	.000			
Continuity Correctionb	31.704	1	.000			
Likelihood Ratio	34.551	1	.000			
Fisher's Exact Test				.000	.000	
Linear-by-Linear Association	32.585	1	.000			
N of Valid Cases	860					
a. 0 cells (.0%) have expected count less than 5. The minimum expected count is 82.25.
b. Computed only for a 2x2 table
	

Functional Dyspepsia *  gender
Crosstab	
	 gender	Total	
	Male	female		
Functional Dyspepsia	No	Count	168	363	531	
		% within  gender	78.1%	56.3%	61.7%	
	Yes	Count	47	282	329	
		% within  gender	21.9%	43.7%	38.3%	
Total	Count	215	645	860	
	% within  gender	100.0%	100.0%	100.0%	


Chi-Square Tests	
	Value	df	Asymp. Sig. (2-sided)	Exact Sig. (2-sided)	Exact Sig. (1-sided)	
Pearson Chi-Square	32.623a	1	.000			
Continuity Correctionb	31.704	1	.000			
Likelihood Ratio	34.551	1	.000			
Fisher's Exact Test				.000	.000	
Linear-by-Linear Association	32.585	1	.000			
N of Valid Cases	860					
a. 0 cells (.0%) have expected count less than 5. The minimum expected count is 82.25.
b. Computed only for a 2x2 table
	
Postprandial distress syndrome *  gender

Crosstab	
	 gender	Total	
	Male	female		
Postprandial distress syndrome	No	Count	199	566	765	
		% within  gender	92.6%	87.8%	89.0%	
	Yes	Count	16	79	95	
		% within  gender	7.4%	12.2%	11.0%	
Total	Count	215	645	860	
	% within  gender	100.0%	100.0%	100.0%	


Chi-Square Tests	
	Value	df	Asymp. Sig. (2-sided)	Exact Sig. (2-sided)	Exact Sig. (1-sided)	
Pearson Chi-Square	3.791a	1	.052			
Continuity Correctionb	3.317	1	.069			
Likelihood Ratio	4.089	1	.043			
Fisher's Exact Test				.059	.031	
Linear-by-Linear Association	3.786	1	.052			
N of Valid Cases	860					
a. 0 cells (.0%) have expected count less than 5. The minimum expected count is 23.75.
b. Computed only for a 2x2 table
	

Epigastric pain syndrome *  gender Crosstabulation	
	 gender	Total	
	Male	female		
Epigastric pain syndrome	No	Count	208	624	832	
		% within  gender	96.7%	96.7%	96.7%	
	Yes	Count	7	21	28	
		% within  gender	3.3%	3.3%	3.3%	
Total	Count	215	645	860	
	% within  gender	100.0%	100.0%	100.0%	


Chi-Square Tests	
	Value	df	Asymp. Sig. (2-sided)	Exact Sig. (2-sided)	Exact Sig. (1-sided)	
Pearson Chi-Square	.000a	1	1.000			
Continuity Correctionb	.000	1	1.000			
Likelihood Ratio	.000	1	1.000			
Fisher's Exact Test				1.000	.574	
Linear-by-Linear Association	.000	1	1.000			
N of Valid Cases	860					
a. 0 cells (.0%) have expected count less than 5. The minimum expected count is 7.00.
b. Computed only for a 2x2 table
	


FBD *  gender
Crosstab	
	 gender	Total	
	Male	female		
FBD	No	Count	156	331	487	
		% within  gender	72.6%	51.3%	56.6%	
	Yes	Count	59	314	373	
		% within  gender	27.4%	48.7%	43.4%	
Total	Count	215	645	860	
	% within  gender	100.0%	100.0%	100.0%	


Chi-Square Tests	
	Value	df	Asymp. Sig. (2-sided)	Exact Sig. (2-sided)	Exact Sig. (1-sided)	
Pearson Chi-Square	29.620a	1	.000			
Continuity Correctionb	28.761	1	.000			
Likelihood Ratio	30.675	1	.000			
Fisher's Exact Test				.000	.000	
Linear-by-Linear Association	29.585	1	.000			
N of Valid Cases	860					
a. 0 cells (.0%) have expected count less than 5. The minimum expected count is 93.25.
b. Computed only for a 2x2 table
	


Irritable Bowel Syndrome *  gender

Crosstab	
	 gender	Total	
	Male	female		
Irritable Bowel Syndrome	No	Count	211	575	786	
		% within  gender	98.1%	89.1%	91.4%	
	Yes	Count	4	70	74	
		% within  gender	1.9%	10.9%	8.6%	
Total	Count	215	645	860	
	% within  gender	100.0%	100.0%	100.0%	


Chi-Square Tests	
	Value	df	Asymp. Sig. (2-sided)	Exact Sig. (2-sided)	Exact Sig. (1-sided)	
Pearson Chi-Square	16.580a	1	.000			
Continuity Correctionb	15.456	1	.000			
Likelihood Ratio	21.648	1	.000			
Fisher's Exact Test				.000	.000	
Linear-by-Linear Association	16.561	1	.000			
N of Valid Cases	860					
a. 0 cells (.0%) have expected count less than 5. The minimum expected count is 18.50.
b. Computed only for a 2x2 table
	


Functional Bloating *  gender
Crosstab	
	 gender	Total	
	Male	female		
Functional Bloating	No	Count	181	414	595	
		% within  gender	84.2%	64.2%	69.2%	
	Yes	Count	34	231	265	
		% within  gender	15.8%	35.8%	30.8%	
Total	Count	215	645	860	
	% within  gender	100.0%	100.0%	100.0%	


Chi-Square Tests	
	Value	df	Asymp. Sig. (2-sided)	Exact Sig. (2-sided)	Exact Sig. (1-sided)	
Pearson Chi-Square	30.255a	1	.000			
Continuity Correctionb	29.324	1	.000			
Likelihood Ratio	33.034	1	.000			
Fisher's Exact Test				.000	.000	
Linear-by-Linear Association	30.220	1	.000			
N of Valid Cases	860					
a. 0 cells (.0%) have expected count less than 5. The minimum expected count is 66.25.
b. Computed only for a 2x2 table
	


Functional Diarrhoea *  gender

Crosstab	
	 gender	Total	
	Male	female		
Functional Diarrhoea	No	Count	214	645	859	
		% within  gender	99.5%	100.0%	99.9%	
	Yes	Count	1	0	1	
		% within  gender	.5%	.0%	.1%	
Total	Count	215	645	860	
	% within  gender	100.0%	100.0%	100.0%	


Chi-Square Tests	
	Value	df	Asymp. Sig. (2-sided)	Exact Sig. (2-sided)	Exact Sig. (1-sided)	
Pearson Chi-Square	3.003a	1	.083			
Continuity Correctionb	.334	1	.563			
Likelihood Ratio	2.776	1	.096			
Fisher's Exact Test				.250	.250	
Linear-by-Linear Association	3.000	1	.083			
N of Valid Cases	860					
a. 2 cells (50.0%) have expected count less than 5. The minimum expected count is .25.
b. Computed only for a 2x2 table
	


Unspecified functional Bowel disorder *  gender
Crosstab	
	 gender	Total	
	Male	female		
Unspecified functional Bowel disorder	No	Count	185	548	733	
		% within  gender	86.0%	85.0%	85.2%	
	Yes	Count	30	97	127	
		% within  gender	14.0%	15.0%	14.8%	
Total	Count	215	645	860	
	% within  gender	100.0%	100.0%	100.0%	


Chi-Square Tests	
	Value	df	Asymp. Sig. (2-sided)	Exact Sig. (2-sided)	Exact Sig. (1-sided)	
Pearson Chi-Square	.151a	1	.698			
Continuity Correctionb	.077	1	.781			
Likelihood Ratio	.152	1	.696			
Fisher's Exact Test				.740	.395	
Linear-by-Linear Association	.151	1	.698			
N of Valid Cases	860					
a. 0 cells (.0%) have expected count less than 5. The minimum expected count is 31.75.
b. Computed only for a 2x2 table
	


Functional abdominal pain syndrome *  gender


Crosstab	
	 gender	Total	
	Male	female		
Functional abdominal pain syndrome	No	Count	203	521	724	
		% within  gender	94.4%	80.8%	84.2%	
	Yes	Count	12	124	136	
		% within  gender	5.6%	19.2%	15.8%	
Total	Count	215	645	860	
	% within  gender	100.0%	100.0%	100.0%	


Chi-Square Tests	
	Value	df	Asymp. Sig. (2-sided)	Exact Sig. (2-sided)	Exact Sig. (1-sided)	
Pearson Chi-Square	22.546a	1	.000			
Continuity Correctionb	21.533	1	.000			
Likelihood Ratio	26.917	1	.000			
Fisher's Exact Test				.000	.000	
Linear-by-Linear Association	22.520	1	.000			
N of Valid Cases	860					
a. 0 cells (.0%) have expected count less than 5. The minimum expected count is 34.00.
b. Computed only for a 2x2 table
	

F. Fecal Incontinence *  gender
Crosstab	
	 gender	Total	
	Male	female		
F. Fecal Incontinence	No	Count	213	638	851	
		% within  gender	99.1%	98.9%	99.0%	
	Yes	Count	2	7	9	
		% within  gender	.9%	1.1%	1.0%	
Total	Count	215	645	860	
	% within  gender	100.0%	100.0%	100.0%	


Chi-Square Tests	
	Value	df	Asymp. Sig. (2-sided)	Exact Sig. (2-sided)	Exact Sig. (1-sided)	
Pearson Chi-Square	.037a	1	.847			
Continuity Correctionb	.000	1	1.000			
Likelihood Ratio	.038	1	.845			
Fisher's Exact Test				1.000	.601	
Linear-by-Linear Association	.037	1	.847			
N of Valid Cases	860					
a. 1 cells (25.0%) have expected count less than 5. The minimum expected count is 2.25.
b. Computed only for a 2x2 table
	


Functional Anorectal disorder *  gender Crosstabulation	
	 gender	Total	
	Male	female		
Functional Anorectal disorder	No	Count	213	638	851	
		% within  gender	99.1%	98.9%	99.0%	
	Yes	Count	2	7	9	
		% within  gender	.9%	1.1%	1.0%	
Total	Count	215	645	860	
	% within  gender	100.0%	100.0%	100.0%	


Chi-Square Tests	
	Value	df	Asymp. Sig. (2-sided)	Exact Sig. (2-sided)	Exact Sig. (1-sided)	
Pearson Chi-Square	.037a	1	.847			
Continuity Correctionb	.000	1	1.000			
Likelihood Ratio	.038	1	.845			
Fisher's Exact Test				1.000	.601	
Linear-by-Linear Association	.037	1	.847			
N of Valid Cases	860					
a. 1 cells (25.0%) have expected count less than 5. The minimum expected count is 2.25.
b. Computed only for a 2x2 table
	
